# Supplementary material for: Data augmentation via warping transforms for modeling natural variability in the corneal endothelium enhances semi-supervised segmentation
Source: PLoS One. 2024 Nov 12;19(11):e0311849. doi: 10.1371/journal.pone.0311849 (PMC11556704; doi:10.1371/journal.pone.0311849)
Supplement: Attachment — Submitted filename: plosone-response-letter.pdf [file pone.0311849.s001.pdf]

PLOS ONE Manuscript: PONE-D-24-00975

Data augmentation via warping transforms for modeling natural variability in the corneal endothelium enhances semi-supervised segmentation

Dear Dr. Belghith,

We are submitting our response letter to the reviewer comments and the revised manuscript. Overall, we appreciate the constructive review, which has led to an improved paper. We want to make the peer review publicly available. Also, we have made the data and the analysis code available on a public repository. The conventions we have used in this letter are:

- *Black text* in italics are editor or reviewer comments
- Blue text are our responses to those comments
- Red text are the new or modified text in the manuscript. When necessary we have included a figure from the paper here or a screenshot with equations.

Regards,

Andrés Marrugo, Ph.D.
Professor of Mechatronics Engineering
Universidad Tecnológica de Bolívar
Cartagena, Colombia

Journal Requirements:

1. When submitting your revision, we need you to address these additional requirements. Please ensure that your manuscript meets PLOS ONE's style requirements, including those for file naming. The PLOS ONE style templates can be found at https://journals.plos.org/plosone/s/file?id=wjVg/PLOSOne_formatting_sample_main_body.pdf and https://journals.plos.org/plosone/s/file?id=ba62/PLOSOne_formatting_sample_title_authors_affiliations.pdf.

RE: The manuscript has been adjusted to the style requirements of PLOS ONE.

2. Please note that PLOS ONE has specific guidelines on code sharing for submissions in which author-generated code underpins the findings in the manuscript. In these cases, all author-generated code must be made available without restrictions upon publication of the work. Please review our guidelines at https://journals.plos.org/plosone/s/materials-and-software-sharing#loc-sharing-code and ensure that your code is shared in a way that follows best practice and facilitates reproducibility and reuse.

RE: The code resulting from the research is available in the following Github repository and the link has been added to the Data availability statement (line 425) at the end of the manuscript: https://github.com/checho9214/Data-augmentation-via-warping---semi-supervised-segmentation

3. Thank you for stating the following financial disclosure: [This work has been partly funded by Ministerio de Ciencia, Tecnología e Innovación, Colombia, Project 124489786239 (Contract 763-2021).]. Please state what role the funders took in the study. If the funders had no role, please state: ""The funders had no role in study design, data collection and analysis, decision to publish, or preparation of the manuscript.""

If this statement is not correct you must amend it as needed. Please include this amended Role of Funder statement in your cover letter; we will change the online submission form on your behalf.

RE: Thank you for the recomendation. We have updated our funding statement on the cover letter and we would like to update our funding statement as follows:

This work has been partly funded by Ministerio de Ciencia, Tecnología e Innovación, Colombia, Project 124489786239 (Contract 763-2021). S. Sanchez thanks Minciencias and Sistema General de Regalías (Programa de Becas de Excelencia) for a PhD scholarship. The funders had no role in study design, data collection and analysis, decision to publish, or preparation of the manuscript.

4. Thank you for stating the following in the Acknowledgments Section of your manuscript: [This work has been partly funded by Ministerio de Ciencia, Tecnologıa e Innovacion, Colombia, Project 124489786239 (Contract 763-2021). S. Sanchez thanks Minciencias and Sistema General de Regal´ıas (Programa de Becas de Excelencia) for a PhD scholarship] We note that you have

provided funding information that is not currently declared in your Funding Statement. However, funding information should not appear in the Acknowledgments section or other areas of your manuscript. We will only publish funding information present in the Funding Statement section of the online submission form. Please remove any funding-related text from the manuscript and let us know how you would like to update your Funding Statement. Currently, your Funding Statement reads as follows: [This work has been partly funded by Ministerio de Ciencia, Tecnología e Innovación, Colombia, Project 124489786239 (Contract 763-2021).]

RE: We have modified the Acknowledgments section accordingly.

5. Your ethics statement should only appear in the Methods section of your manuscript. If your ethics statement is written in any section besides the Methods, please move it to the Methods section and delete it from any other section. Please ensure that your ethics statement is included in your manuscript, as the ethics statement entered into the online submission form will not be published alongside your manuscript.

RE: Thanks for your recomendation. We have moved the ethics statement to the methods section in line 213.

6. This study explores advancements in the segmentation of corneal endothelium images through a novel data augmentation approach. The authors propose a semi-supervised learning framework that integrates unsupervised data with a smaller subset of labeled data to improve segmentation accuracy. Utilizing a combination of models including DenseNet121, and a unique warping transformation technique, the paper claims to outperform standard augmentation methods. While the findings are significant as they offer the potential for more detailed segmentation, the paper requires major revisions in data interpretation and evaluation of results.

RE: Thank you for your recommendation. We have added more details on related work (page 5) for a more nuanced data interpretation and evaluation of results, and have added further discussion and interpretation in pages 12 and 13 with an additional performance metric (AUROC).

Additional Editor Comments

Related work:

1. *Since, the article aims to enhance the segmentation algorithm by improving data augmentation, it is recommended that the authors provide this with numerical results from previous related works for clearer benchmarking in this section (AUC, ACC and Dice)*

RE: Thank you for this suggestion. We have added other performance metrics and have also reported these metrics as a benchmark for our proposed method. Nevertheless, it is important to note that most work in the literature reports performance based on healthy cells. We added this analysis and discussion in page 13. We finish this discussion with the following:

Our method surpasses these benchmarks, particularly in handling the variability and challenges posed by diseased cells, demonstrating the efficacy of incorporating data augmentation via warping transforms to enhance semi-supervised segmentation tasks.

Dataset description and Table 1:

2. *The authors should specify the number of unique patients and eyes in each dataset (training, testing, and validation). Additionally, details on the distribution of healthy and pathological corneas in these sets would be valuable.*

RE: Thank you for your recommendation. We have revised the data description to the following in page 6:

A set of 90 in vivo specular microscopy images of endothelial cells in two-channel TIF format, with a resolution of 640x480 pixels, obtained from 66 patients with healthy (42 from the right eye) and dystrophic (48 from the left eye) corneas were used. The data was split by patient, it is important to note that there was no overlap of patient images in the training, testing and validation sets. From these images, 271 patches of size 96x96 were extracted that were labeled by expert personnel, and 1719 patches of the same dimensions without annotations, with balanced distributions for all possible cases. Images of the same patient are not repeated in the training set, nor in the test and validation sets. Images were acquired with a Topcon SP3000P specular microscope equipped with Cell Count software. The study protocol received approval from the ethics committee of the Technological University of Bolívar in Colombia. Due to the retrospective approach of the study, the requirement to obtain informed consent was not applicable. Furthermore, the study was carried out in accordance with the principles established in the Declaration of Helsinki.

3. *Authors should clarify whether data was split by image, eye, or patient level and confirm whether there is any overlap of images from the same patient across the training, testing, and validation datasets.*

RE: Thank you for your recommendation. The data were divided by patients, a set of 90 in vivo specular microscopy images of endothelial cells in two-channel TIF format, with a resolution of 640x480 pixels, obtained from 66 patients with healthy corneas (42 from the right eye) and dystrophic corneas (48 of the left eye). In the case of the images of diseased cells with Fuchs' dystrophy, low, medium and high levels of gutta were observed, distributed from the center of the cornea to the periphery. Importantly, there was no overlap of patient images in the training, testing, and validation sets.

4. *Table 1: The allocation of data into 216 training, 25 testing, and 30 validation samples deviates from conventional norms, particularly with the validation set being larger than the testing set. Could the authors provide a rationale for this distribution?*

RE: Thank you for your recommendation. The distribution of samples in Table 1 reflects careful consideration of our data's nature and our study's objectives. We assigned more samples to the validation set than the testing set to fine-tune model hyperparameters during training. This choice helps improve model performance on unseen data and prevents overfitting to the training set. We acknowledge the regulations and the importance of testing data to obtain an unbiased estimate and verify the model's performance in practice. The difference of 5 images between the validation and testing stages is not significant, but validation is crucial for fine-tuning the model and avoiding overfitting.

5. *Table 1: The article describes using data augmentation to increase the sample size. Typically, augmentation is applied uniformly across all images, and they are either replaced or combined with the original images, resulting in the augmented dataset being an integer multiple of the original dataset. However, Table 1 shows that the train data increased from 1719 to 4220 (a 2.76 times increase) and from 216 to 597 (a 2.45 times increase) for the unsupervised and supervised sections, respectively. This is not consistent with an integer multiple increase. The authors need to clarify whether the augmentation was applied selectively to parts of the data multiple times or if another method was used.*

RE: Thank you for your recommendation. During data augmentation, we generated different deformations for each image by adjusting the parameter "s" to values greater than zero. Distortions larger than zero were more plausible and could be selected, while higher distortions often rendered images implausible and unusable. Therefore, the augmentation process was not uniform. This resulted in the training data increasing from 1719 to 4220 (a 2.76-fold increase) for the unsupervised section and from 216 to 597 (a 2.45-fold increase) for the supervised section.

We applied data augmentation in different proportions to the unsupervised and supervised stages. The unsupervised stage (Barlow twins) required greater variability to capture the diversity of features, hence the higher augmentation. In the supervised stage, where segmentation is performed, a less pronounced augmentation was applied to avoid overfitting and ensure proper generalization to unseen data.

Semi Supervised Model and Architecture:

6. *The authors should provide clearer details about the unsupervised model within the semi-supervised framework shown in Figure 2. Specifically, clarify whether Models ResNet50, ResNet101, DenseNet121, and ResNet101ViT are used as encoders in the unsupervised section, and confirm if DenseNet121 is the only encoder used in the supervised section, or if the same models are employed across both sections. Please revise this section (and Figure 2 if necessary) for greater clarity.*

RE: In response to this critique, we propose a weakly supervised model implemented in two stages. In the first stage, unsupervised learning uses different backbones (ResNet50, ResNet101,

DenseNet121, and ResNet101ViT) as encoders pre-trained with a Siamese network based on the Barlow Twins method. This stage aims to learn feature representations without data annotations. In the second stage, these pre-trained weights are frozen, and fine-tuning is performed with supervised learning to learn the segmentation task using a few images of the corneal endothelium. Below is the block diagram of the proposed architecture.

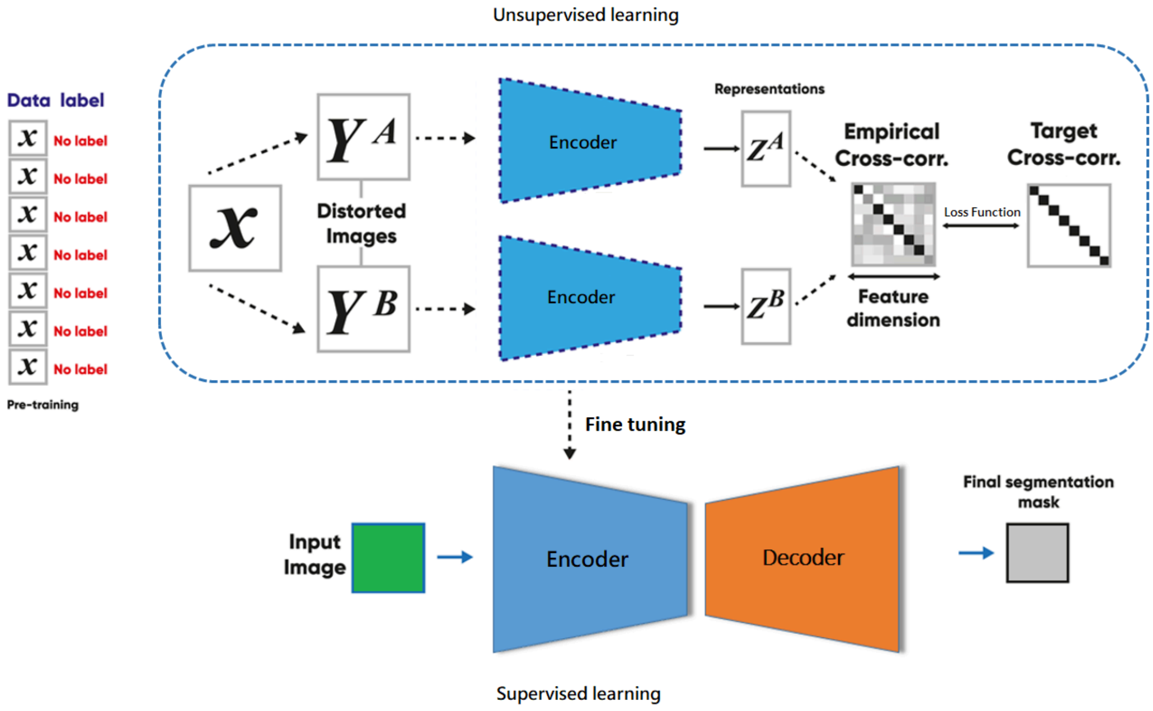

Figure 2: *Block diagram of the proposed semi-supervised model*

Data Augmentation section:

7. *In Figure 3, during the keypoints extraction phase, the method extracts points at equal distances along all borders of the image (including all four sides and each square). However, these points do not appear to be centered on any cell. As a result, some cells, especially on the edges, have two very closely spaced centers identified, one of which may be incorrect. This redundancy could significantly impact the performance of the Delaunay triangulation. Could the authors please clarify this issue?*

RE: Thank you for your recommendation. The extraction of closely spaced keypoints near the image edges prevents unrealistic transformations, such as black triangles. While this approach limits the maximum deformation for the image, it still allows for the generation of new training images. This method ensures deformations are constrained to avoid unrealistic

transformations, yet sufficient to produce augmented training samples. We added the following comment in page 9:

The extraction of closely spaced keypoints near the image edges prevents unrealistic transformations, such as black triangles, as illustrated in fig. 3. This approach ensures deformations are constrained to avoid unrealistic artifacts while still producing augmented training samples.

8. *In The section "Warping through Local Affine Transformations" discusses the parameter and its influence on image deformation, yet is absent from the formulas provided. Additionally, it is unclear which numerical values of correspond to the data in Table 1 (are these values from high- or low-deformation categories used in the training dataset?). The authors need to specify the numerical values of used in Table 1 and Figure 4 to help readers understand this parameter's impact and efficacy.*

RE: Thank you for pointing this out. We have further explained how the warping parameter s in the manuscript and have updated the table and figure to clarify the values used and the impact in the final transformation as follows:

Original keypoints K are modified to create a new set of keypoints K', facilitating the generation of a new triangulation grid for the warped image (Fig. 3). Each keypoint with coordinates (x_i, y_i) in K is shifted by random amounts to produce the new keypoints K' given by

$$K' = \{(x'_i, y'_i) \mid x'_i = x_i + \delta x,\ y'_i = y_i + \delta y\}\,, \tag{3}$$

where δx and δy are random shifts. Let R be a random variable following a uniform distribution in $(-1, 1) \in \mathbb{R}$, a random shift, δ, is then computed as follows,

$$\delta = \frac{R \cdot d_{min}}{s}, \tag{4}$$

where $d_{\min}$ is the minimun distance between any two points in K defined as

$$d_{\min} = \min_{a,b \in K, a \neq b} d(a, b), \tag{5}$$

and

$$d(a, b) = \min(|x_a - x_b|, |y_a - y_b|). \tag{6}$$

The parameter s in Equation 4 inversely controls the magnitude of the transformation: a smaller s results in larger shifts and consequently more pronounced warping [69].

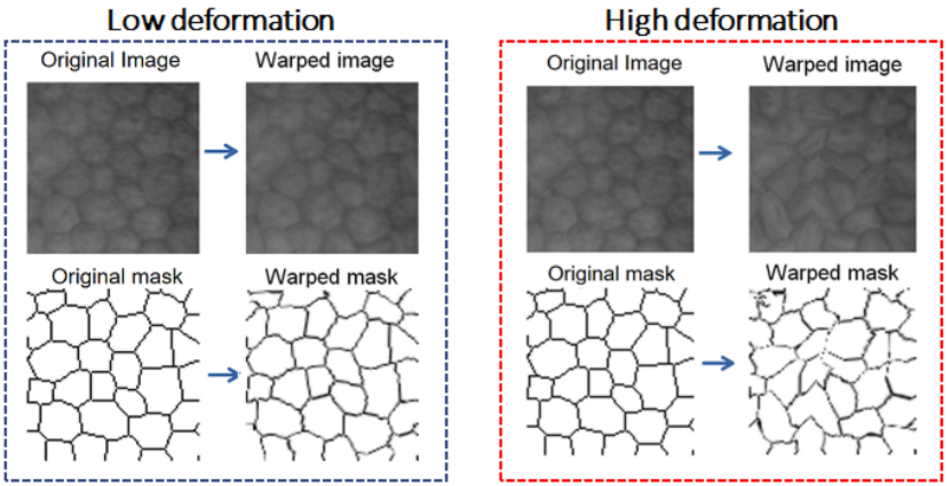

Fig 4. Transformation via warping with low ($s = 3$) and high ($s = 2$) deformation levels.

Results and conclusions:

9. *The names of the models prefixed with "BT-" in Table 2 and Figure 8 are not defined in the text. Please clarify what "BT-" stands for.*

   RE: Thank you for your recommendation. BT is the contraction of the word Barlow Twins. The legend of this meaning was added in figure 8.

10. *The authors should include the AUROC as a metric in table 2, alongside ACC. AUROC is more informative for segmenting corneal endothelium images, where class imbalances are common (most pixels being non-border, class 0) and can provide a more comprehensive evaluation of the model's performance across various thresholds.*

RE: Thank you for this suggestion. We have included the AUROC metric and all models were trained again to compute the performance metrics obtaining satisfactory results compared to the state of the art. As can be seen in the following table. This has been described further in page 13.

Model		Strategy 1				Strategy 2				Strategy 3			
		Auroc	Acc	Dc	mIoU	Auroc	Acc	Dc	mIoU	Auroc	Acc	Dc	mIoU
**BT-UNet**		0.7	0.69	0.78	0.60	0.81	0.81	0.84	0.66	**0.88**	**0.873**	**0.85**	**0.725**
**BT-ResNet50**		0.78	0.78	0.868	0.65	0.86	0.84	0.85	0.67	**0.9**	**0.912**	**0.913**	**0.838**
**BT-ResNet101**		0.63	0.63	0.87	0.66	0.83	0.84	0.86	0.65	**0.87**	**0.897**	**0.91**	**0.85**
**BT-ResNet101ViT**		0.78	0.76	0.82	0.67	0.83	0.83	0.84	0.68	**0.85**	**0.845**	**0.89**	**0.766**
**BT-DenseNet121**		0.81	0.81	0.86	0.67	0.88	0.88	0.87	0.69	**0.926**	**0.915**	**0.94**	**0.86**

From the above table, we can see that the data augmentation strategy with warping method and semi-supervised model is an excellent option when little labeled medical data is available. This approach shows good performance for both imaging healthy cells and those with Fuchs dystrophy. Strategy 3 presented the best performance in the AUROC, ACC, DC and mIoU metrics.

11. *The authors should compare and discuss their results with those of other methods that have shown superior performance in this domain. For example, the study linked here https://pubmed.ncbi.nlm.nih.gov/29680687/ achieved an AUROC of 0.92 using conventional augmentation methods.*

RE: We have responded to this critique in our previous answer.

12. *The authors should add the results from common augmentation methods to Table 2 for comparison with the proposed method. This will help determine how much of the improvement is due to the unique and effective augmentation approach introduced in the article.*

RE: Thank you for your comment. All training strategies included conventional augmentation. However, only the baseline (strategy 1) used exclusively conventional augmentation (horizontal flip, vertical flip, and rotations). This distinction allows us to demonstrate that strategies 2 and 3 outperform conventional augmentation. Additionally, strategy 3 shows the best performance because it includes augmentation via warping in the supervised stage as well. We have clarified this on page 12.
