# Supplementary material for: Data augmentation via warping transforms for modeling natural variability in the corneal endothelium enhances semi-supervised segmentation
Source: PLoS One. 2024 Nov 12;19(11):e0311849. doi: 10.1371/journal.pone.0311849 (PMC11556704; doi:10.1371/journal.pone.0311849)
Supplement: Attachment — Submitted filename: plosone-response-letter-second-round.pdf [file pone.0311849.s003.pdf]

PLOS ONE Manuscript: PONE-D-24-00975

Data augmentation via warping transforms for modeling natural variability in the corneal endothelium enhances semi-supervised segmentation

Response to Comments/Suggestions of Reviewers

We express our gratitude for the time and effort dedicated to reviewing our manuscript. The suggestions and comments allowed us to improve it. We worked diligently to address all the concerns raised by the reviewers. Below we provide our detailed responses to critiques. Please note that unless otherwise indicated, page numbers, figures, references, and tables in the modified/added text refer to those in the revised document.

- Reviewer comments are in *Black italics*.
- Our responses to critiques are in Blue.
- New or modified contents are in Red.

Regards,

Andrés Marrugo, Ph.D.
Professor of Mechatronics Engineering
Universidad Tecnológica de Bolívar
Cartagena, Colombia

Journal Requirements:

Please review your reference list to ensure that it is complete and correct. If you have cited papers that have been retracted, please include the rationale for doing so in the manuscript text, or remove these references and replace them with relevant current references. Any changes to the reference list should be mentioned in the rebuttal letter that accompanies your revised manuscript. If you need to cite a retracted article, indicate the article's retracted status in the References list and also include a citation and full reference for the retraction notice.

RE: Our reference list is complete and correct. During our revision several citations have changed order.

Reviewer #1

Comment 1: *The authors are encouraged to succinctly summarize the newly added related work, focusing particularly on the essential details relevant to the study, as cited in References 57-59.*

RE: We appreciate your suggestion. We have summarized these paragraphs further.

Fabijańska et al. \cite{PMID:29680687} used a U-NET architecture to segment corneal endothelial images, achieving an AUROC of 0.92 and a DICE coefficient of 0.86, indicating high precision in boundary delineation. Similarly, Nurzynska (2018) applied CNNs for automatic cell segmentation, reaching 93\% accuracy compared to manual annotations and a modified Hausdorff distance of 0.14 pixels, demonstrating strong segmentation performance. Hao et al. \cite{QU2022142} developed a deep learning system for estimating morphometric parameters and segmenting corneal endothelial cells from in vivo confocal microscopy images. Tested on 99 subjects, it achieved a Pearson correlation of 0.932 (\$p<0.01\$) with Topcon cell density measurements and an AUC of 0.923, surpassing a U-Net model with an AUC of 0.913.

Shilpashree et al. \cite{shilpashree2021automated} compared average perimeter length (APL) and endothelial cell density (ECD) between Fuchs' endothelial dystrophy (FECD) patients and healthy subjects. Using U-Net and Watershed for segmentation, they found decreased ECD and increased APL with higher guttae in FECD, indicating endothelial deterioration. For healthy subjects, the method achieved an F1 score of 82.27\%, an average IoU of 77.27\%, a precision of 87.9\%, and an ROC AUC of 96.70\%, demonstrating high segmentation accuracy.

Comment 2: *Please ensure that all abbreviations are clearly defined at their first occurrence within the manuscript. For example, "The UW approach" has not been defined, which I presume might refer to 'U-Net and Watershed.' This abbreviation should be explicitly clarified to maintain clarity for all readers.*

RE: Thank you for the recommendation. We have verified that all abbreviations are defined. The "UW approach" has been rewritten to avoid the unnecessary acronym.

Comment 3: *While the added "Dataset Description" section is informative, Table 1 currently lacks sufficient details regarding the distribution of data across the train,*

validation, and test sets. I recommend enhancing Table 1 with additional columns to clarify the following aspects:

Patient Distribution: Out of the 66 unique patients in this study, please specify how many patients belong to the healthy category and how many to the dystrophic category.
Dataset Breakdown: For each category (healthy and dystrophic), provide details on the number of unique patients included in the train, validation, and test datasets (e.g., the training set includes 23 unique patients with 17 being healthy and 6 dystrophic).
Specular Microscopy Images: Apply the same detailed breakdown to the 90 in vivo specular microscopy images regarding their distribution across the train, validation, and test datasets.
Additionally, please revise the newly added paragraph to reflect these details for enhanced clarity and completeness.

RE: Thank you for your valuable suggestion. In response, we have enhanced Table 1 to include detailed information regarding the distribution of unique patients (healthy and dystrophic) and the breakdown of the images across the training, validation, and test datasets.

Table 1: Data distribution for training, testing, and validation of the semi-supervised model.

Strategy	Unsupervised Stage (No Labels)	State of Cells in Unlabeled Images	Number of Patients (No Labels)	Supervised Stage (Labels)	State of Cells in Labeled Images	Number of Patients (Labels)
1 (Baseline)	1719	859 (healthy cells)<br>860 (cells with dystrophy)	29 patients (right eye)<br>29 patients (left eye)	271<br>(216 for training, 25 for test, 30 for validation)	Training:<br>108 (healthy cells)<br>108 (cells with dystrophy)<br>Test:<br>12 (healthy cells)<br>13 (cells with dystrophy)<br>Validation:<br>15 (healthy cells)<br>15 (cells with dystrophy)	Training:<br>4 patients (right eye)<br>4 patients (left eye)<br>Test:<br>1 patient (right eye)<br>1 patient (left eye)<br>Validation:<br>1 patient (right eye)<br>1 patient (left eye)
2	4220	2110 (healthy cells)<br>2110 (cells with dystrophy)	29 patients (right eye)<br>29 patients (left eye)	271<br>(216 for training, 25 for test, 30 for validation)	Training:<br>108 (healthy cells)<br>108 (cells with dystrophy)<br>Test:<br>12 (healthy cells)<br>13 (cells with dystrophy)<br>Validation:<br>15 (healthy cells)<br>15 (cells with dystrophy)	Training:<br>4 patients (right eye)<br>4 patients (left eye)<br>Test:<br>1 patient (right eye)<br>1 patient (left eye)<br>Validation:<br>1 patient (right eye)<br>1 patient (left eye)
3	4220	2110 (healthy cells)<br>2110 (cells with dystrophy)	29 patients (right eye)<br>29 patients (left eye)	652<br>(597 for training, 25 for test, 30 for validation)	Training:<br>298 (healthy cells)<br>299 (cells with dystrophy)<br>Test:<br>12 (healthy cells)<br>13 (cells with dystrophy)<br>Validation:<br>15 (healthy cells)<br>15 (cells with dystrophy)	Training:<br>10 patients (right eye)<br>10 patients (left eye)<br>Test:<br>1 patient (right eye)<br>1 patient (left eye)<br>Validation:<br>1 patient (right eye)<br>1 patient (left eye)

Comment 4: *Please revise and incorporate the main points from your following responses in "Author's Response To Reviewer Comments" file (answers for different comments) into the discussion section of your manuscript:*

4.1:
"The distribution of samples in Table 1 reflects careful consideration of our data's nature and our study's objectives. We assigned more samples to the validation set than the testing set to fine-tune model hyperparameters during training. This choice helps improve model performance on unseen data and prevents overfitting to the training set. We acknowledge the regulations and the importance of testing data to obtain an unbiased estimate and verify the model's performance in practice."
4.2:

"During data augmentation, we generated different deformations for each image by adjusting the parameter "s" to values greater than zero. Distortions larger than zero were more plausible and could be selected, while higher distortions often rendered images implausible and unusable. Therefore, the augmentation process was not uniform. This resulted in the training data increasing from 1719 to 4220 (a 2.76-fold increase) for the unsupervised section and from 216 to 597 (a 2.45-fold increase) for the supervised section.
We applied data augmentation in different proportions to the unsupervised and supervised stages. The unsupervised stage (Barlow twins) required greater variability to capture the diversity of features, hence the higher augmentation. In the supervised stage, where segmentation is performed, a less pronounced augmentation was applied to avoid overfitting and ensure proper generalization to unseen data."

RE: Thank you for your valuable suggestions. In response, the key points from our previous responses to comments 4.1 and 4.2 have been carefully reviewed and incorporated into the discussion section of the manuscript. These revisions address the distribution of samples and the data augmentation process, ensuring that the rationale and methodology are clearly explained.

The sample distribution in Table 1 was designed with careful consideration of the data and study objectives. More samples were allocated to the validation set than the test set to fine-tune hyperparameters during training. This approach improves performance on unseen data and helps prevent overfitting. We also recognize the importance of the test set for obtaining an unbiased estimate of model performance.

During data augmentation, deformations were applied by adjusting the parameter "s" to values greater than zero. Plausible distortions were selected, while higher distortions rendered images unusable, resulting in non-uniform augmentation. This increased the unsupervised training data from 1719 to 4220 (2.76-fold) and the supervised data from 216 to 597 (2.45-fold). Greater augmentation was applied in the unsupervised stage (Barlow twins) to capture feature diversity, while less augmentation was used in the supervised stage to avoid overfitting and ensure generalization.

Comment 5: The authors' response regarding the "Semi-Supervised Model and Architecture" is notably concise and effectively clarifies the methods used in their research, despite being significantly shorter than the corresponding section in the manuscript. I recommend revising the "Architectures" section to mirror this clarity and conciseness, which will enhance the overall readability and precision of the manuscript.

RE: Thank you for your thoughtful recommendation. We have carefully revised the "Architecture" and "Semi-supervised Model" sections, incorporating the suggestions provided to improve clarity and alignment with the reviewer's feedback.

\subsection*{Semi-supervised model}

We propose a semi-supervised model consisting of two stages. In the first stage, unsupervised learning is used to train various encoders, including ResNet50, ResNet101, DenseNet121, and ResNet101ViT, within a Siamese network architecture based on the Barlow Twins method \cite{zbontar2021barlow}. This process learns feature representations from a large set of unlabeled images. In the second stage, the encoder weights obtained from the first stage are frozen, and fine-tuning is performed with supervised learning to segment corneal endothelial images using a small number of labeled images.

As shown in Figure \ref{fig:bd-ssl-models}, the model processes unlabeled images (X) through a Siamese network, where each image undergoes geometric transformations (e.g., rotations, flipping, cropping) to generate new samples (Y^A and Y^B). These transformed images are passed through identical encoders to produce feature maps (Z^A and Z^B), and consistent patterns are identified using the cross-correlation function. The learned weights are then fixed and applied during fine-tuning. A decoder with attention modules and skip connections is added, and a 1×1 convolution at the output generates the final segmentation mask.

\subsubsection*{Architectures}

We use state-of-the-art pretrained backbones—ResNet50 \cite{he2015deep}, ResNet101 \cite{lee2017wideresidualinception}, DenseNet121 \cite{huang2018densely}, and ResNet101ViT \cite{chen2022vision}—within our semi-supervised learning framework. These models, trained on large-scale datasets, have proven effective at learning robust features that enhance performance in our corneal endothelial image segmentation task.

ResNet architectures are used for their deep learning capabilities and residual blocks that prevent performance degradation in deep networks. DenseNet is employed for its dense layer connections, allowing the model to capture detailed and contextual features. Finally, ResNet101ViT, which combines ResNet with Vision Transformer (ViT), captures both local and global features by applying attention mechanisms and processing images in patches \cite{app12188972}. This combination improves feature extraction and segmentation accuracy in medical imaging tasks.

Our approach demonstrates significant improvements in model generalization across different encoders, regardless of the pretrained backbone used.
