# Supplementary material for: Data augmentation via warping transforms for modeling natural variability in the corneal endothelium enhances semi-supervised segmentation
Source: PLoS One. 2024 Nov 12;19(11):e0311849. doi: 10.1371/journal.pone.0311849 (PMC11556704; doi:10.1371/journal.pone.0311849)
Supplement: Attachment — Submitted filename: Review_JJ.docx [file pone.0311849.s002.docx]

**Comment 1:** The authors are encouraged to succinctly summarize the newly added related work, focusing particularly on the essential details relevant to the study, as cited in References 57-59.

**Comment 2:** Please ensure that all abbreviations are clearly defined at their first occurrence within the manuscript. For example, "The UW approach" has not been defined, which I presume might refer to 'U-Net and Watershed.' This abbreviation should be explicitly clarified to maintain clarity for all readers.

**Comment 3:** While the added "Dataset Description" section is informative, Table 1 currently lacks sufficient details regarding the distribution of data across the train, validation, and test sets. I recommend enhancing Table 1 with additional columns to clarify the following aspects:

Patient Distribution: Out of the 66 unique patients in this study, please specify how many patients belong to the healthy category and how many to the dystrophic category.

Dataset Breakdown: For each category (healthy and dystrophic), provide details on the number of unique patients included in the train, validation, and test datasets (e.g., the training set includes 23 unique patients with 17 being healthy and 6 dystrophic).

Specular Microscopy Images: Apply the same detailed breakdown to the 90 in vivo specular microscopy images regarding their distribution across the train, validation, and test datasets.

Additionally, please revise the newly added paragraph to reflect these details for enhanced clarity and completeness.

**Comment 4:** Please revise and incorporate the main points from your following responses in "Author's Response To Reviewer Comments" file (answers for different comments) into the discussion section of your manuscript:

4.1:

"The distribution of samples in Table 1 reflects careful consideration of our data's nature and our study's objectives. We assigned more samples to the validation set than the testing set to fine-tune model hyperparameters during training. This choice helps improve model performance on unseen data and prevents overfitting to the training set. We acknowledge the regulations and the importance of testing data to obtain an unbiased estimate and verify the model's performance in practice."

4.2:

"During data augmentation, we generated different deformations for each image by adjusting the parameter "s" to values greater than zero. Distortions larger than zero were more plausible and could be selected, while higher distortions often rendered images implausible and unusable. Therefore, the augmentation process was not uniform. This resulted in the training data increasing from 1719 to 4220 (a 2.76-fold increase) for the unsupervised section and from 216 to 597 (a 2.45-fold increase) for the supervised section.

We applied data augmentation in different proportions to the unsupervised and supervised stages. The unsupervised stage (Barlow twins) required greater variability to capture the diversity of features, hence the higher augmentation. In the supervised stage, where segmentation is performed, a less pronounced augmentation was applied to avoid overfitting and ensure proper generalization to unseen data."

**Comment 5:** The authors' response regarding the "Semi-Supervised Model and Architecture" is notably concise and effectively clarifies the methods used in their research, despite being significantly shorter than the corresponding section in the manuscript. I recommend revising the "Architectures" section to mirror this clarity and conciseness, which will enhance the overall readability and precision of the manuscript.
